# T-cadherin deficiency increases vascular vulnerability in T2DM through impaired NO bioactivity

**DOI:** 10.1186/s12933-016-0488-0

**Published:** 2017-01-19

**Authors:** Han Wang, Ling Tao, Anastasia Ambrosio, Wenjun Yan, Ross Summer, Wayne Bond Lau, Yajing Wang, Xinliang Ma

**Affiliations:** 1 Department of Cardiology, Xijing Hospital, Fourth Military Medical University, 147 West Changle Rd, Xi’an, 710032 Shaanxi China; 2 Department of Emergency Medicine, Thomas Jefferson University, 1025 Walnut Street, 808 College Building, Philadelphia, PA 19107 USA; 3 Department of Medicine, Thomas Jefferson University, 1025 Walnut Street, College Building, Philadelphia, PA 19107 USA

**Keywords:** Endothelial cell, Endothelial dysfunction, T-cadherin, T2DM, NO bioactivity, Vascular ring

## Abstract

**Background:**

Endothelial dysfunction plays a critical role in the development of type 2 diabetes (T2DM). T-cadherin (T-cad) has gained recognition as a regulator of endothelial cell (EC) function. The present study examined whether T-cad deficiency increases vascular vulnerability in T2DM.

**Methods:**

Vascular segments were isolated from WT or T-cad knockout mice. Endothelial function, total NO accumulation, and the expression of T-cad related proteins were determined.

**Results:**

Ach and acidified NaNO2 induced similar vasorelaxation in WT groups. T-cad KO mice exhibited normal response to acidified NaNO2, but manifested markedly reduced response to Ach. NO accumulation was also decreased in T-cad KO group. T-cad expression was reduced in WT mice fed 8 weeks of high fat diet (HFD). Furthermore, exacerbated reduction of vasorelaxation was observed in T-cad KO mice fed 8 weeks of HFD.

**Conclusions:**

In the current study, we provide the first in vivo evidence that T-cadherin deficiency causes endothelial dysfunction in T2DM vascular segments, suggesting the involvement of T-cad deficiency in T2DM pathogenesis.

## Background

Type 2 diabetes mellitus (T2DM) affects approximately 100 million people worldwide [1]. Diabetic vascular complications are responsible for the majority of morbidity and mortality in the diabetic population. These complications can be arbitrarily divided into micro- and macrovascular complications. Macrovascular complications are associated with accelerated atherosclerosis, resulting in premature ischemic heart disease, increased risk of cerebrovascular disease, and severe peripheral vascular disease [2].

Endothelial dysfunction is characterized by deficiency of nitric oxide (NO) production in response to normal secretion signals. A critical component of atherosclerosis development, endothelial dysfunction is a characteristic abnormality observed in diabetes [3–5]. The bioavailability of NO, produced by endothelial NO synthase (eNOS), represents a key marker of vascular health. Down-regulated NO may contribute to atherogenic predisposition. Many metabolic derangement factors cause endothelial dysfunction by affecting the balance of NO production during diabetes development [6–8]. Therefore, clarification of the mechanisms responsible for endothelial dysfunction in diabetes, and identification of the therapeutic interventions that may improve endothelial function hold great potential in reducing cardiovascular complications and overall death in diabetic patients.

T-cadherin (T-cad) is a unique member of the cadherin family. Found on the cellular surface, it possesses no intracellular domain. T-cadherin content is maximal in the aorta, carotid, iliac, and renal arteries, and in the heart. Known to regulate neuronal growth during embryogenesis, T-cad has additionally gained recognition as a regulator of endothelial cell (EC) function [9–11].

In vivo expression of T-cad is increased in human atherosclerotic lesions and experimental restenosis. In vitro expression of T-cad is upregulated in proliferating endothelial and smooth muscle cells, as well as endothelial cells during oxidative and endoplasmic reticulum stress [11, 12]. Clinical evidence underlines the association between T-cad and hypoadiponectinemia, with increased risk of various metabolic diseases [13, 14]. Together, these observations strongly suggest the involvement of T-cad in diabetic vasculopathic state development. However, direct evidence that supported T-cadherin deficiency exists in diabetic endothelial dysfunction pathogenesis is currently lacking.

Therefore, the aims of the present study were: (1) to determine whether T-cad deficiency is involved in the development of T2DM, (2) to determine whether T-cad deficiency may cause endothelial dysfunction in descending aortic vascular segments (the most frequent location of atherosclerosis development), (3) to determine whether endothelial dysfunction is exacerbated by T-cad deficiency in descending aortic vascular segments in T2DM, and (4) to elucidate the responsible underlying mechanisms.

## Methods

### Determination of vasorelaxation in aortic tissue

Adult male T-cad knockout mice (T-cad KO) or their wild type littermates (WT) were used in all study experiments. All experiments were performed in adherence to the NIH Guidelines on the Use of Laboratory Animals, and approved by the Thomas Jefferson University Committee on Animal Care. After animals were anesthetized with 3% isoflurane, descending aortas were removed and placed into cold Krebs buffer solution [NaCl 118, KCl 4.75, CaCl_2_·2H_2_O 2.54, KH_2_PO_4_ 1.19, MgSO_4_·7H_2_O 1.19, NaHCO_3_ 25, and glucose 10.0 (mM)] Aorta were carefully cut into 2–3 segments (each 2–3 mm length); surrounding fat and tissue were debrided. Segments were suspended upon stainless steel hooks, and aerated (95% O_2_ and 5% CO_2_) in 37 °C 5 ml K–H tissue baths. Aortic rings were connected to FORT-10 force transducers (WPI, Sarasota, FL) for MacLab data acquisition. Segments were first stretched to generate 2.5 mN force, followed by 0.5 mN increments every 15 min until achieving 4 mN total force. As force was increased to 8mN, the Krebs buffer solution was replaced with a buffer with increased potassium content (HK solution) for vessel ring sustainability. Once 8 mN force was achieved, the tissue bath was replaced with normal Krebs buffer. During the period of force decrease from 8 to 4 mN, epinephrine (a potent vasoconstrictor) was added to the tissue bath in 2 concentrations (10^−5^ and 10^−4^ M) in sequence within 2 min of each other, to induce vasoconstriction. After ~5 min, the vasorelaxant acetylcholine (concentrations of 10^−6^, 10^−5^, 10^−4^, 10^−3^ and 10^−2^ M) were added to the tissue bath in immediate succession. The percent relaxation from the peak of the epinephrine-induced contraction to the nadir point of acetylcholine-induced relaxation was recorded on MacLab.

### Determination of NO accumulation from aortic segments

To determine total NO accumulation, isolated aortic segments were placed in culture medium and incubated in a cell culture incubator (5% CO_2_, 37 °C). After 8 h of incubation, segments were subjected to homogenization. Protein concentrations were determined via BCA method (Pierce Chemical). Medium was transferred into the wells of 96-well plate, and incubated with nitrate reductase and cofactors for 20 min at 37 °C to reduce NO_3_ to NO_2_. Samples (50 μl) were then injected into a water-jacketed, oxygen-free purge vessel containing 5 ml of 20 mM potassium iodide in glacial acetic acid to reduce NO_2_ to NO. Resultant chemiluminescence from the reaction of NO and ozone was detected by a nitric oxide analyzer (SIEVERS NOA 280I; Sievers, Boulder, CO). Detector signals were collected and analyzed by a PC-based data recording and processing system. To determine the NO content of the culture medium, calculations of the slope of the regression analysis were performed. The amount of NO_x_ released was expressed in nmol/mg protein.

### Determination of NO_x_ production from aortic segments

To detect endothelial NO_x_ production, isolated aortic segments were placed in 6-well plates with 500 μl culture medium. Epinephrine (10^−4^ M) was added to each well. 3 min later, acetylcholine concentrations (10^−6^, 10^−5^, 10^−4^, 10^−3^ and 10^−2^ M) were added in immediate succession. Resultant medium nitrite (NO_2_^−^) and nitrate (NO_3_^−^) levels were determined by chemiluminescence NO detector (Siever 280i NO Analyzer).

### Quantitation of tissue nitrotyrosine content

Nitrotyrosine content, the accepted footprint of in vivo peroxynitrite (ONOO^−^) formation, was determined by ELISA described in our previous publication [15]. In brief, aortic segments were homogenized in lysis buffer and centrifuged for 10 min at 12,000*g* at 4 °C. Supernatants were collected and protein concentrations were determined. Tissue samples from aortic segments, were applied to disposable sterile ELISA plates, and incubated overnight with primary antibody against nitrotyrosine (05–233, Millipore, USA). After extensive wash and incubation with the peroxidase-conjugated secondary antibody, the peroxidase reaction product was generated using TMB solution. The optical density was measured at 450 nm with a SpectraMax-Plus microplate spectrophotometer. The amount of nitrotyrosine content in tissue samples was calculated using standard curves generated from nitrated BSA containing known amounts of nitrotyrosine, and expressed as pmol/mg protein.

### Immunoblotting

Aortic tissue homogenate proteins were separated on SDS-PAGE gels, transferred to PVDF membranes, and Western blotted with monoclonal antibody against eNOS, Ser^1177^ phosphorylated eNOS (Becton–Dickinson, USA), and polyclonal antibody against Akt and Ser^473^ phosphorylated Akt (Cell Signaling Technology, USA). PVDF membranes were incubated with horseradish peroxide-conjugated anti-rabbit or anti-mouse IgG antibodies (Cell Signaling Technology, USA) for 2 h. The blot was developed using Super-Signal Reagent (Pierce) and visualized with a Kodak Image Station 4000R. Blot densities were analyzed (Gelpro32 software).

### Real-time PCR

Total RNA was extracted from aortic segments via RNeasy Mini Kit (QIAGEN, USA). Expression analysis of the reported genes was performed by real-time PCR via commercial kit (4367659, AB) and the ABI 7500 Sequence Detection System, using SYBR GREEN as a double-stranded DNA-specific dye. GAPDH served as endogenous control. Table 1 lists the primers for mRNA expression analysis by real-time PCR.

**Table 1:** Primers sequences for Real-time PCR analysis

Genes	Primers
GAPDH-F (mouse)	5′-AGGTCGGTGTGAACGGATTTG-3′
GAPDH-R (mouse)	5′-TGTAGACCATGTAGTTGAGGTCA-3′
T-cad-F (mouse)	5′-CATCGAAGCTCAAGATATGG-3′
T-cad-R (mouse)	5′-GATTTCCATTGATGATGGTG-3′

### Statistical analysis

All values in the text, table, and figures are presented as mean ± SEM of n independent experiments. Data (except Western blot density) were subjected to *t* test (two groups) or ANOVA (three or more groups) followed by Bonferoni correction for post hoc *t* test. Western blot densities were analyzed by the Kruskal–Wallis test followed by Dunn’s post test. Probabilities of 0.05 or less were considered to be statistically significant.

## Results

### T-cad mRNA and protein expression decreased in WT mice fed 8 weeks HFD

**Fig. 1:**
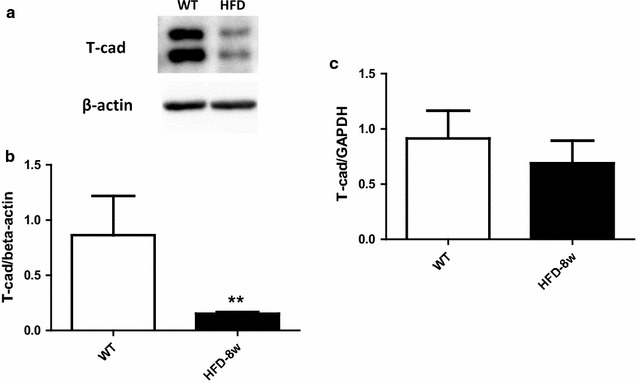
T-cad mRNA and protein expression were reduced in WT mice fed 8 weeks HFD. T-cad expression as measured by Western blot and real time PCR **a** representative *Western blots*; **b** *Western blot* density analysis (n = 4); **c** Real time PCR analysis (n = 3).**P < 0.01 vs. WT

We first determined T-cad expression during T2DM condition. In an established diet-induced T2DM model, we fed WT mice high fat diet for 8 weeks, and determined both the mRNA and protein levels of T-cadherin [16]. Both mRNA and protein levels of T-cadherin were reduced compared to control, suggesting T-cadherin deficiency occurs during type 2 diabetes (Fig. 1).

### T-cad deficiency induced endothelial dysfunction

**Fig. 2:**
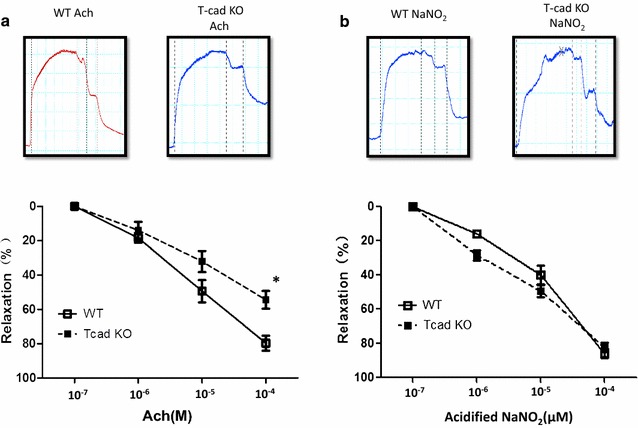
T-cad deficiency induced endothelial dysfunction. Concentration-dependent vasorelaxation of aortic vascular rings in response to a Ach, an endothelium-dependent vasodilator, and b acidified NaNO_2_, an endothelium-independent vasodilator. *Top* representative figures; *Bottom* analysis result. Each mouse aorta generated 2–3 rings, n = 8–12 rings/group. *WT* wild type litternates, *T*-*cad KO* T-cadherin knockout mice. *P < 0.05 vs. WT

We next performed a vascular ring experiment to directly address this relationship. In WT aortic rings, administration of Ach (an endothelial-dependent NO donor) resulted in concentration-dependent vasorelaxation, with no significant difference compared to the effects elicited by acidified NaNO_2_ (an endothelial-independent NO donor). At maximal concentration (100 μM), Ach and acidified NaNO_2_ induced 79.7 and 85.9% vasorelaxation respectively, demonstrating preserved vascular ring endothelial function post-preparation protocols. However, aortic rings from T-cad KO mice exhibited markedly reduced vasorelaxation by Ach (endothelial-dependent stimulation) treatment compared to that induced by acidified NaNO_2_ (endothelial-independent NO donor) treatment. Furthermore, addition of 100 μM Ach resulted in only 54.5% relaxation (P < 0.01 vs. WT) in T-cad KO aortic ring; 100 μM acidified NaNO_2_ induced 82.3% relaxation (P > 0.05 vs. WT) (Fig. 2). Together, these results demonstrate T-cad deficiency resulted in severe endothelial dysfunction in the tested aortic segments.

### T-cad deficiency-induced endothelial dysfunction exacerbated in T2DM

**Fig. 3:**
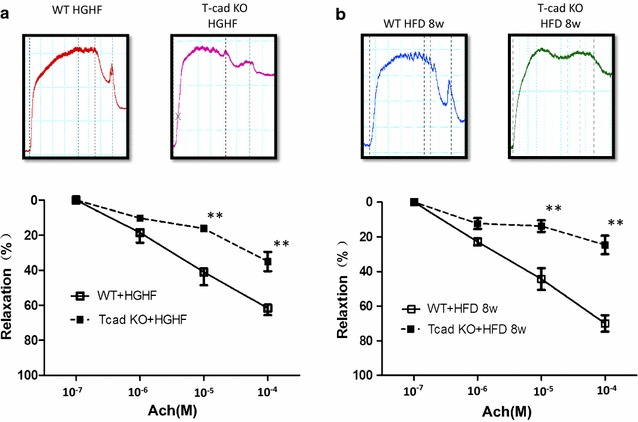
T-cad deficiency induced-endothelial dysfunction was exacerbated in T2DM. Concentration-dependent vasorelaxation of aortic vascular rings subjected to **a** high glucose/high fat treatment (high glucose: 25.5 m mol/l; high fat/palmitate: 300 μ mol/l) and **b** high fat diet alone for 8 weeks. *Top* representative figures; *Bottom* analysis result. Each mouse aorta generated 2–3 rings, n = 6–7 rings/group. *WT* Wild type litternates, *T*-*cad KO* T-cadherin knockout mice. **P < 0.01 vs. WT

The above experiments verified T-cad deficiency occurs in T2DM, and T-cad deficiency was associated with vascular segment endothelial dysfunction. Next, we determined the state of endothelial function in T-cad KO animals under in-viro or in vivo diabetic environment. We first utilized an in vitro model of diabetes, employing high glucose (HG, 25.5 mmol/l), high fat (HF, palmitate 300 μmol/l) treatment. After incubating aortic rings from WT and T-cad KO mice in HGHF (2.5 h), vasorelaxation was impaired in both animal types. However, the reduction in concentration-dependent vasorelaxation was markedly increased in T-cad KO mice compared to WT. To better mimic in vivo T2DM, we next fed WT mice and T-cad KO mice 8 weeks of HFD diet as previously described [17]. Again, ring vasorelaxation was reduced in both WT and T-cad KO mice. Vasorelaxation reduction was more pronounced in T-cad KO mice compared to WT mice (Fig. 3). Collectively, these results suggest that endothelial dysfunction caused by T-cad deficiency was exacerbated in the T2DM condition.

### Total NO accumulation and NO_X_ production significantly decreased in vascular segments from T-cad KO animals

**Fig. 4:**
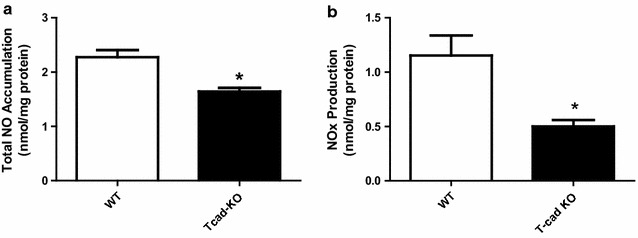
Basal NO production was significantly decreased in vascular segments from T-cad deficient animals. **a** NO concentration in culture medium after 8 h incubation of aortic segments from WT or T-cadherin knockout mice. **b** Concentration of nitric oxide in medium after Ach stimulation. Each mouse aorta generated 2 aortic segments. n = 8 segments/group. *P < 0.05 vs. WT

Next, total NO accumulation and NO_x_ production were determined. Aortic segments were isolated from WT and T-cad KO animals, and incubated with cell culture medium. Culture media was collected after 8 h. Total NO accumulation in aortic segments from T-cad KO mice was significantly decreased compared to WT (Fig. 4). To measure direct NO release after Ach stimulation, total NO_x_ production was determined. As expected, total NO_x_ production was markedly decreased in T-cad KO mice compared to WT. Together, these results demonstrate that T-cad deficiency was associated with reduced NO production, which may be responsible in part for the observed state of endothelial dysfunction.

### Phosphorylation of Akt, but not eNOS, was significantly reduced in T-cad KO aortic tissue

**Fig. 5:**
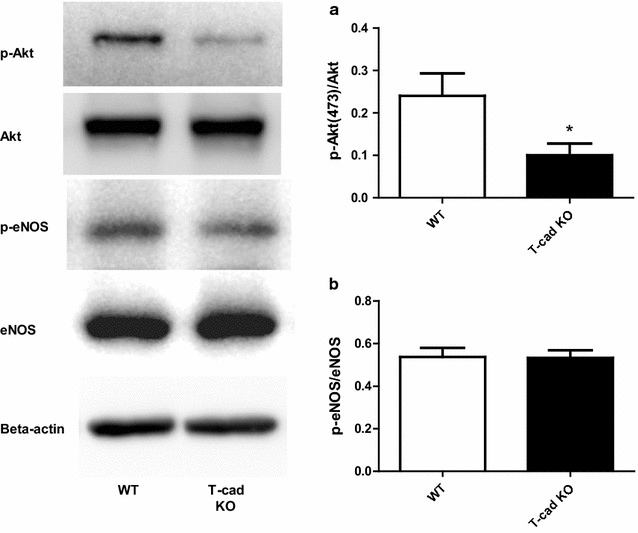
Phosphorylation of Akt, but not eNOS, was significantly reduced in aortic tissue from T-cad KO animals. Expression of Akt, p-Akt, eNOS and p-eNOS in aortic tissues obtained from WT or T-cadherin knockout mice. *Left* representative *Western blots*. *Right* summary of density analysis of p-Akt/Akt (**a**) and p-eNOS/eNOS (**b**), n = 3 mice/group, *P < 0.05 vs. WT

To study the underlying mechanisms, we performed Western blot. Akt phosphorylation was significantly increased in T-cad KO mice compared to WT (Fig. 5), consistent with known current research. However, eNOS phosphorylation in T-cad KO mice was slightly increased (not to significant degree) compared to WT. These results suggest that reduction of NO bioavailability caused by T-cad deficiency may induce phosphorylation of the Akt-eNOS pathways in compensatory manner, without significant effect.

### Akt inactivation increases caspase-3 activity in HUVECs

**Fig. 6:**
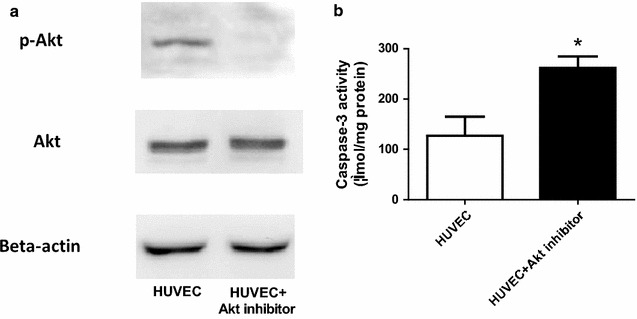
Akt inactivation increased caspase-3 activity in HUVECs. **a** Representative *Western blots* of p-Akt and Akt expression in HUVECs with or without Akt inhibitor; **b** Caspase-3 activity, using Ac-DEVD-pNA as substrate, n = 3, *P < 0.05 vs. WT

To further investigate the contributive mechanisms of reduced NO bioactivity, we cultured and subjected human umbilical vein endothelial cells (HUVECs) to Akt inhibitor (A6730, Sigma, (10 μM) treatment for 24 h. Akt phosphorylation and caspase-3 activity were determined. Akt phosphorylation was significantly decreased, whereas caspase-3 activity was elevated in HUVECs subjected to Akt inhibitor treatment compared to control (Fig. 6). These results strongly suggest Akt inhibition-induced endothelial cell apoptosis contributes to reduced NO bioactivity in T-cad KO aortic tissue.

### Increased nitric oxide inactivation exacerbated endothelial dysfunction in T-cad KO mice

**Fig. 7:**
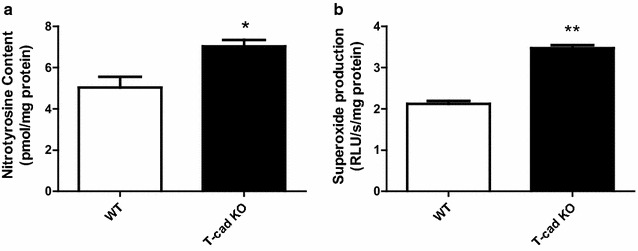
Increased nitric oxide inactivation exacerbated endothelial dysfunction in T-cad KO mice. **a** Aortic nitrotyrosine content determined by ELISA; **b** quantitative assay of superoxide production determined by lucigenin-enhanced chemiluminescence, n = 3–4 mice/group, *P < 0.05 vs. WT, **P < 0.01 vs. WT

We next determined whether the endothelial dysfunction observed in T-cad deficient animals is related to increased superoxide production and nitric oxide inactivation. Tissue nitrotyrosine level was determined, the accepted in vivo footprint of peroxynitrite (ONOO^−^), the product of NO and superoxide. Superoxide production was markedly increased in T-cad KO animals, and nitrotyrosine was significantly increased in T-cad KO mice compared to WT (Fig. 7). These results support augmented NO inactivation as a primary cause of endothelial dysfunction in T-cad deficient animals.

## Discussion

Adiponectin (APN) is a multifunctional adipocytokine of adipose tissue origin. APN exists in three isoforms (trimer, hexamer, and multimer). Plasma APN concentration (which consists of mostly the latter two high molecular weight isoforms) highly correlates with the development of T2DM. Numerous studies have shown that plasma adiponectin level is decreased in individuals with T2DM, indicating APN plays an important role in pathogenesis of T2DM [18–20]. T-cad has been identified as an important receptor of APN, particularly for the high molecular weight isoform [21–24]. Both plasma APN concentration and tissue APN expression are closely related with T-cad [14]. Additionally, T-cad itself is implicated with the pathogenesis of T2DM, but with uncertain role [25, 26]. In the present study, we have provided the first in vivo evidence that T-cad expression is decreased in the aorta from T2DM animals (Fig. 1). This data is consistent with Matsuda’s study demonstrating T-cad expression is positively correlated with plasma APN concentration, which is markedly decreased in T2DM patients [14].

Atherosclerosis is the basis for the vascular pathologies of T2DM. Endothelial dysfunction plays a key role in this process, and the ongoing underlying mechanisms [27–29]. Many studies have demonstrated T-cad plays a central multifunctional role in the vascular system [30–34]. Heretofore, no direct in vivo functional evidence exists demonstrating the role T-cad plays in endothelial dysfunction. We next performed a vascular ring experiment to study the relationship between T-cad and endothelial dysfunction. Our results demonstrated significantly reduced vasorelaxation in T-cad KO mice compared to WT, consistent with numerous in vitro studies (Fig. 2) [35, 36], suggesting T-cad deficiency may cause severe endothelial dysfunction. To investigate whether T2DM exacerbates endothelial dysfunction already caused by T-cad deficiency, we fed mice high-fat diet for 8 weeks to mimic T2DM. Our resultant data provide the first in vivo functional evidence T-cad deficiency exacerbates severe endothelial dysfunction in the T2DM condition (Fig. 3).

NO, produced by eNOS, is a critical vasodilator in the vascular system. NO bioavailability is a surrogate marker of endothelial cell function. To obtain further evidence supporting endothelial dysfunction occurs in T-cad KO vascular segments, we next performed total NO accumulation and NO_x_ production. Our results confirmed that NO production and NO_x_ production were both reduced in T-cad deficient animals (Fig. 4), suggesting T-cad deficiency was associated with reduced NO production, which may be responsible in part for the observed state of endothelial dysfunction.

Interestingly, phosphorylation of Akt was significantly decreased in T-cad KO animals, while phosphorylation of eNOS was grossly unchanged compared to WT (Fig. 5). At the beginning, we thought this reduction in Akt phosphorylation may induce the reduction in eNOS phosphorylation, which is a classical factor to mediate NO production [37]. But our eNOS results, which are consistent with the data of others, did not support this hypothesis [9]. The PI3 k/Akt pathway is highly associated with apoptosis [38]. The reduction in Akt phosphorylation may be contributive to apoptosis induction in EC, which partially explains why NO and NOx production were reduced in EC in vascular segments in T-cad KO mice. Therefore, we utilized Akt inhibitor to attenuate Akt phosphorylation and found caspase-3 activity was significantly increased in HUVECs subjected to Akt inhibitor (Fig. 6). Collectively, our results, which is consistent with the data of others [9, 35], suggested Akt inhibition induced apoptosis, but not reduced eNOS phosphorylation, contributes to decreased NO_x_ production in ECs. Further studies directly addressing this phenomenon are expected.

Experimental and clinical data have demonstrated augmented superoxide production and the resultant superoxide/nitric oxide bi-radical reaction is the primary cause for NO inactivation under pathological conditions. Newly produced NO is oxidized to NO_2_^−^ and NO_3_^−^ rapidly under physiological conditions, reducing NO bio-availability and its vasorelaxative potential, termed NO inactivation. In the presence of superoxide, NO forms peroxynitrite (ONOO^−^), a toxic molecule that modifies tyrosine residues in proteins to create nitrotyrosine, leaving a footprint detectable in vivo [39]. In our present study, we have demonstrated both increased superoxide and nitrotyrosine content in T-cad KO animals (Fig. 7).

## Conclusions

We have provided the first in vivo evidence that T-cad deficiency causes endothelial dysfunction in T2DM model vascular segments, supporting T-cad deficiency involvement in the pathogenesis of T2DM complications. Supplanting T-cad deficiency may be a potential therapeutic avenue in the prevention and amelioration of vascular injury in the diabetic population.

## Abbreviations

Ac-DEVD-pNA: *N*-Acetyl-Asp-Glu-Val-Asp-*p*-Nitroanilide
Ach: acetylcholine
APN: adiponectin
BCA: bicinchoninic acid
EC: endothelial cell
eNOS: endothelial NO synthase
GAPDH: glyceraldehyde 3-phosphate dehydrogenase
HF: high fat
HG: high glucose
HFD: high fat diet
NO: nitric oxide
NO_2_^−^: nitrite
NO_3_^−^: nitrate
ONOO^−^: peroxynitrite
T2DM: type 2 diabetes mellitus
T-cad: T-cadherin
T-cad KO: T-cadherin knockout
WT: wild type
